# Supplementary material for: Anomalous Thermal Characteristics of Poly(ionic liquids) Derived from 1-Butyl-2,3-dimethyl-4-vinylimidazolium Salts
Source: Polymers (Basel). 2022 Jan 8;14(2):254. doi: 10.3390/polym14020254 (PMC8781894; doi:10.3390/polym14020254)
Supplement: Supplementary file 1 [file polymers-14-00254-s001.zip › polymers-1532658-supplementary.pdf]

Anomalous Thermal Characteristics of Poly(ionic liquids) Derived from 1-Butyl-2,3-Dimethyl-4-Vinylimidazolium Salts

Fan Yang[i]*, Meng Zhao*[ii]*, Darren Smith*[iii]*, Peggy Cebe*[iv]*, Sam Lucisano*[v]*, Thomas Allston*[vi]*, and Thomas W. Smith,*[vi] *

Versick Analytics, [i] Jersey City, NJ

BOE Technology Group, [ii] Beijing China

Experimental Therapeutics Branch, Walter Reed Army Institute of Research, [iii]

Silver Spring, MD 20910

Physics and Astronomy Department, Tufts University, [iv] Medford, MA, 02155

Xerox Corporation, [v] Webster, NY

School of Chemistry & Materials Science, Rochester Institute of Technology [vi]

**Correspondence to: twssch@rit.edu*

Supplementary Information

Thermal gravimetric analysis (TGA) was carried out under a nitrogen atmosphere with a TA Instruments TGA 2050. The temperature was increased from 25-600°C at 20°C/min under a nitrogen atmosphere, and then held at 600°C for 10 minutes under an air atmosphere. TGA mass loss profiles for triflate and $TFSI^-$ salts of poly(1-butyl-2,3-dimethylimidazolium) are shown in Figure S1. The thermal stability of the triflate and $TFSI^-$ salts is similar. Significant mass loss in the $TFSI^-$ salt occurs between 430 and 505 °C; that in the triflate salt occurs between 440 and 505°C.

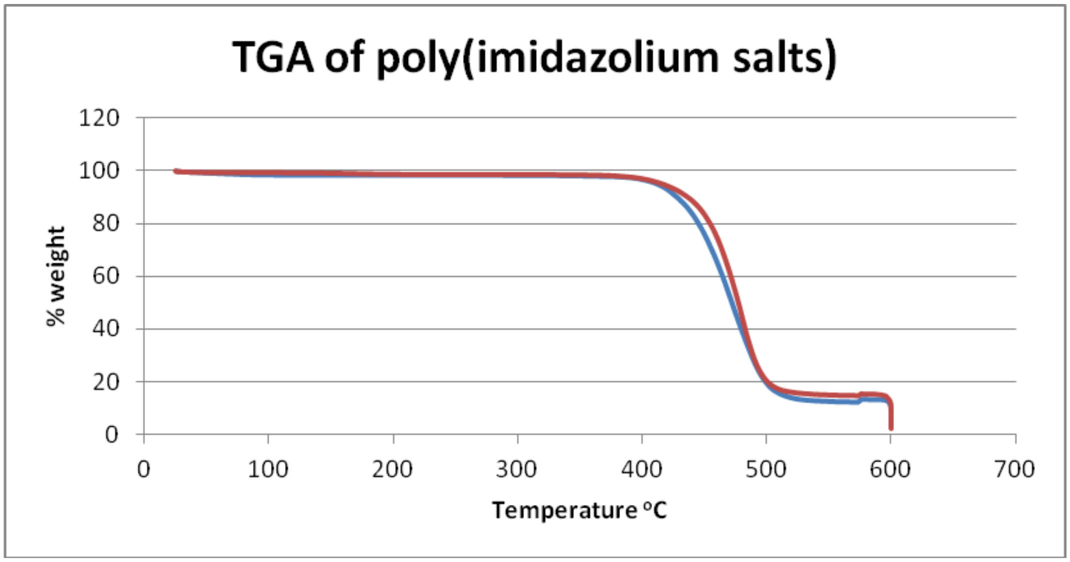

Figure S1 - TGA of poly(1-butyl-2,3-dimethyl-4-vinylimidazolium triflate)(red) and poly(1-butyl-2,3-dimethyl-4-vinylimidazolium TFSI)(blue).

Glass transition characteristics of poly(1-butyl-2,3-dimethyl-4-vinylimidazoliium) triflate, hexaflurophosphate and trifluoromethylsulfonylimide salts were evaluated by differential scanning calorimetry. DSC thermograms for the triflate polymer are shown in **Figure S2** where plots of change in heat capacity in 20 °C/min heating and cooling cycles and an auxiliary 40 °C/min rapid heating cycle are shown.

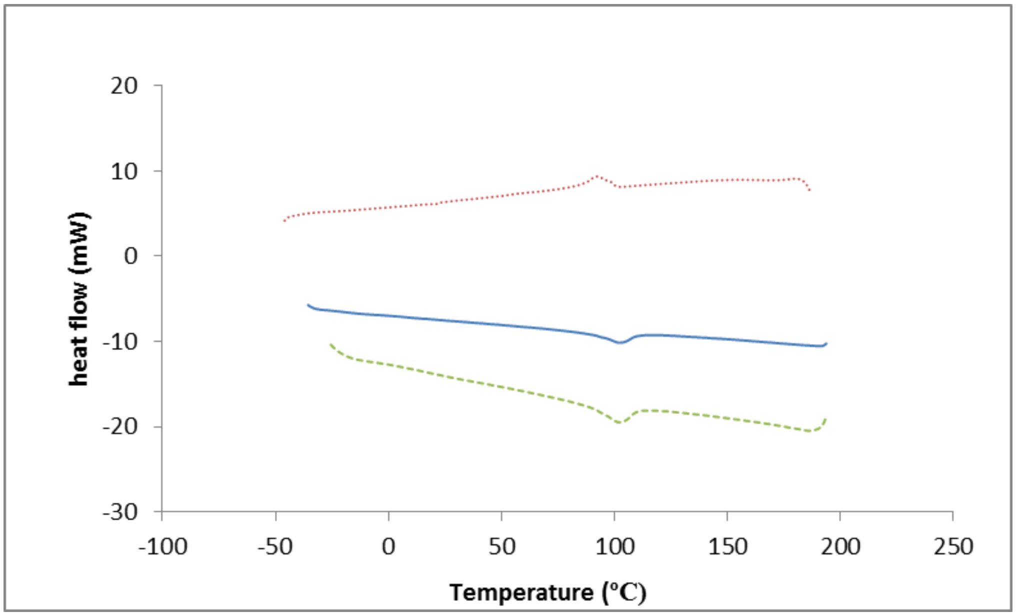

Figure S2 - DSC scans of P23DMVIm^{+} Tf^{-} : solid blue line (20°C/min – heating cycle), dotted red line (20°C/min – cooling cycle), dashed green line (40°C/min – rapid heating cycle).

Thermograms for the TFSI and hexafluorophosphate polymer salts are displayed in Figure S3 and **S4**, respectively.

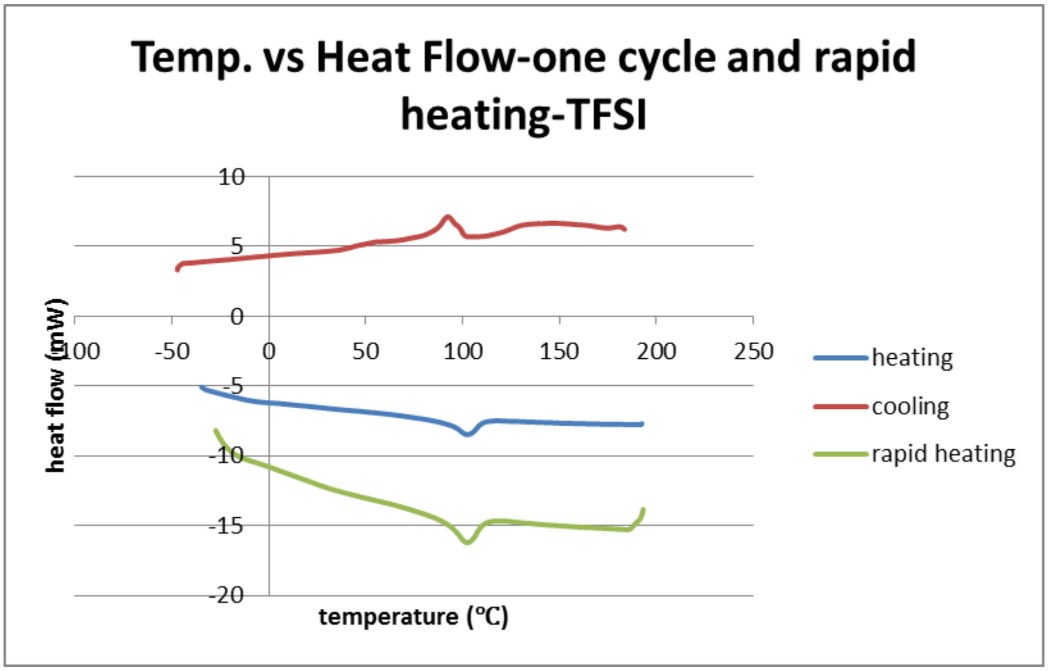

Figure S3 - DSC scans of P23DMVIm^{+}TFSI^{-} during heating and subsequent cooling. The green line is from a final heating cycle at 40° per min.

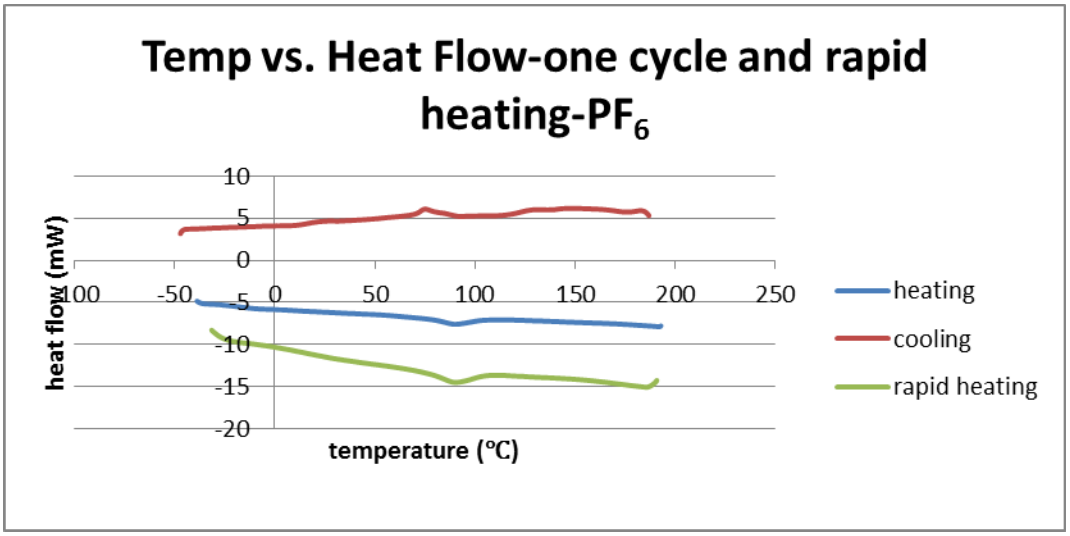

Figure S4 - DSC scans of P23DMVIm$^+$PF$_6^-$: solid blue line (20°C/min – heating cycle), dotted red line (20°C/min – cooling cycle), dashed green line (40°C/min – rapid heating cycle).

The TFSI$^-$ salt exhibited a T_g of 96ºC – 102ºC in the heating cycle and 92 ºC – 84 ºC in the cooling cycle. The hexafluorophosphate anion is less solvating and smaller than TFSI$^-$, yet it exhibits a somewhat lower T_g than that of the TFSI$^-$ salt.

The ^{1}H NMR of 2-methyl-4-vinylimidazole is shown in **Figure S5**.

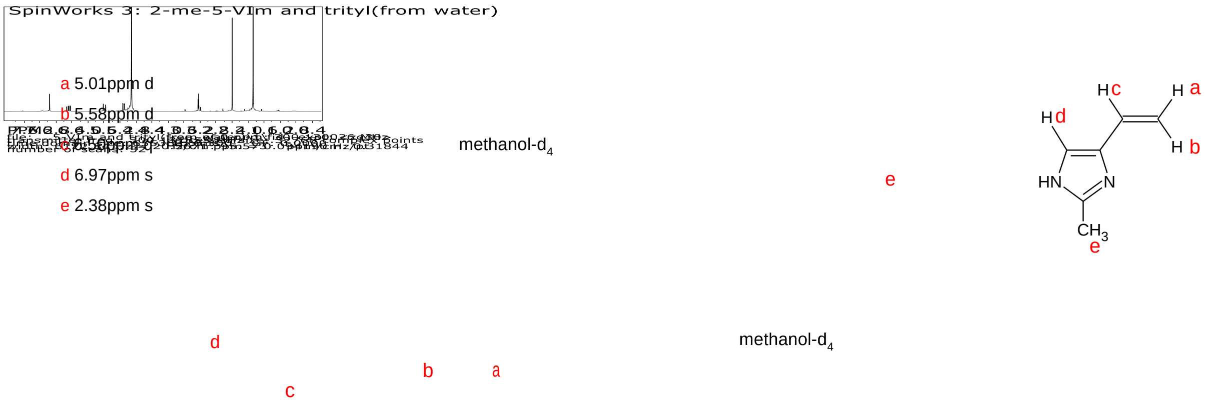

Figure S5. ^{1}H NMR of 2-methyl-4-vinylimidazole

Ha and Hb are in different chemical environments and they are coupled with each other and with Hc to yield split doublets. Hc is also a pair of split doublets, a result of coupling to both Ha and Hb. Hd on the imidazole ring shows a singlet at 6.97ppm and the methyl hydrogens at the 2-position exhibit a chemical shift of 2.38ppm. The sharp singlet at around 1.9 ppm is due to residual cyclohexane, which was the solvent of n-butyl lithium.

The ^{1}H NMR of 1-butyl-2-methyl-4-vinylimidazole is shown in **Figure S6**.

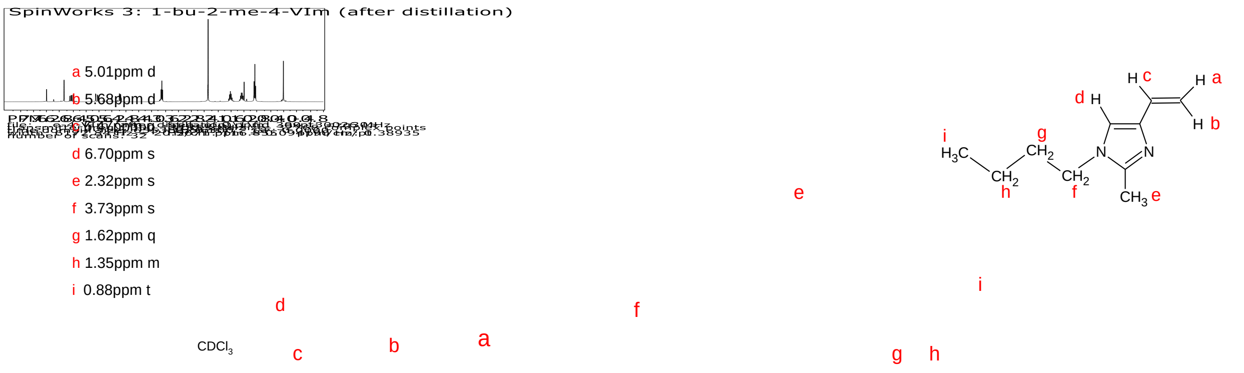

Figure S6. ^{1}H NMR of 1-butyl-2-methyl-4-vinylimidazole

The ^{1}H NMR of 1-butyl-2,3-dimethyl-4-vinylimidazolium triflate is shown in **Figure S7**. The 3-position methyl hydrogen has a chemical shift of 3.73ppm. Hf is more up field than Hg because hydrogens on the methyl group are shielded by the cone of the pi-cloud of the vinyl group.

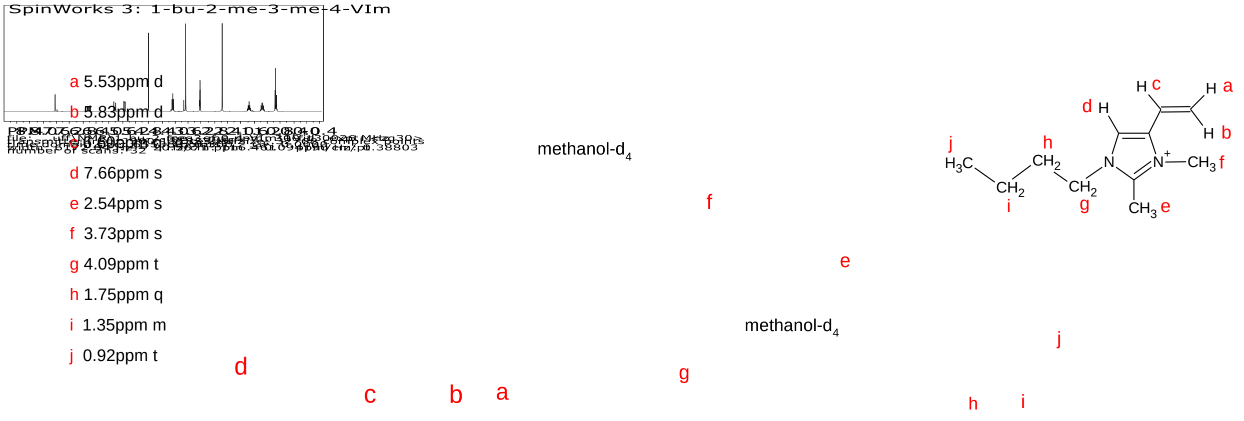

Figure S7. ^{1}H NMR of 1-butyl-2,3-dimethyl-4-vinylimidazolium triflate

The ^{1}H NMR spectrum of Poly(1-butyl-2,3-dimethyl-4-vinylimidazolium) triflate is displayed in **Figure S8**

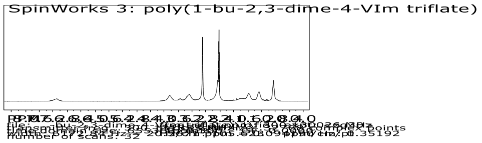

Figure S8 ^{1}H NMR spectrum of Poly(1-butyl-2,3-dimethyl-4-vinylimidazolium) triflate

Poly(1-butyl-2,3-dimethyl-4-vinylimidazolium triflate) was ion-exchanged with $(CF_3SO_2)_2N^-$ and PF_6^-. The efficacy of the ion-exchange process described in the Experimental Section of the paper was verified by the comparative ^{19}F NMR spectra of poly(1-butyl-2,3-dimethyl-4-vinylimidazolium triflate) (**Figure S9**) and poly(1-butyl-2,3-dimethyl-4-vinylimidazolium hexafluorophosphate) **(Figure S10)**, respectively, in which the ^{19}F NMR spectrum of the fully ion-exchanged hexafluorophosphate polymer shows no trace of a peak at ~78 ppm corresponding to ^{19}F in $CF_3SO_3^-$.

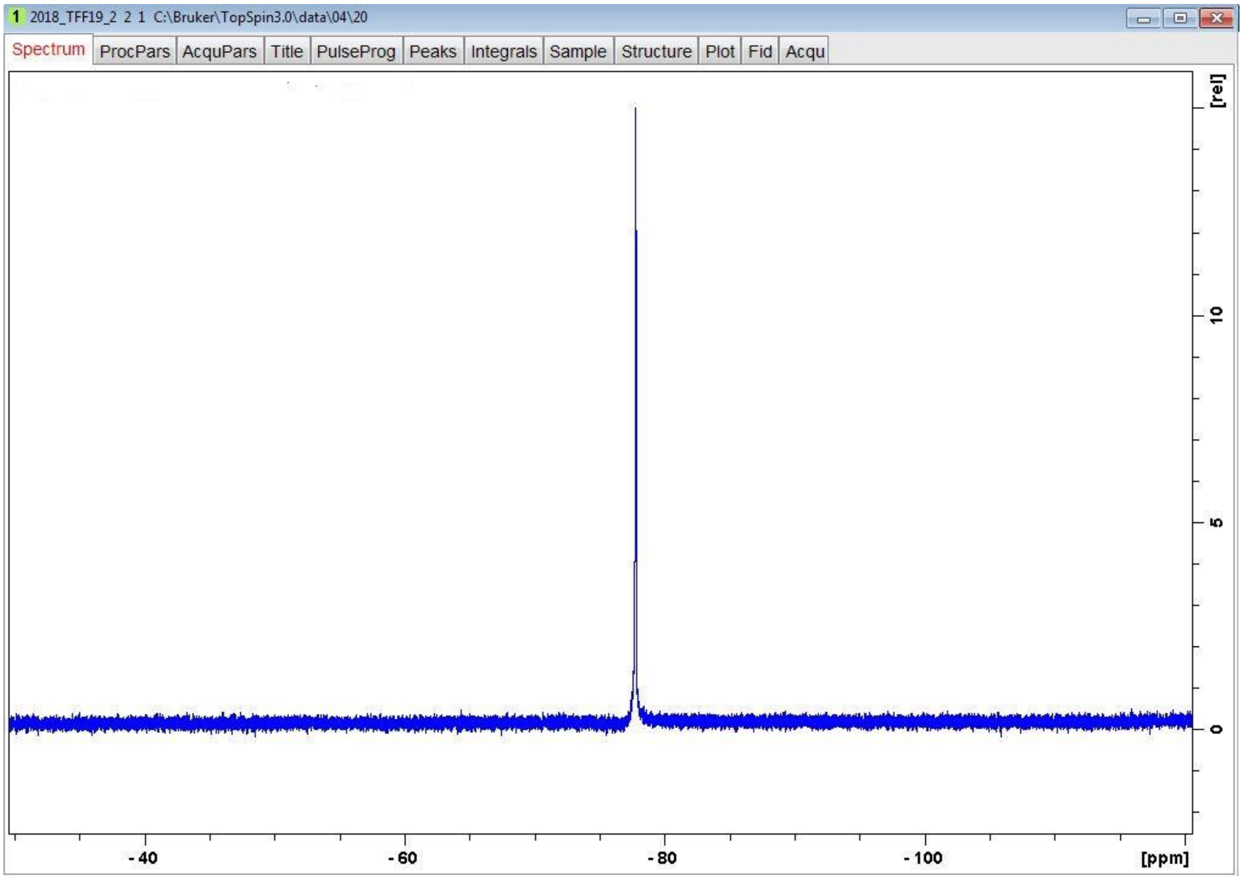

Figure S9 - ^{19}F NMR spectrum of poly(1-butyl-2,3-dimethyl-4-vinylimidazolium triflate)

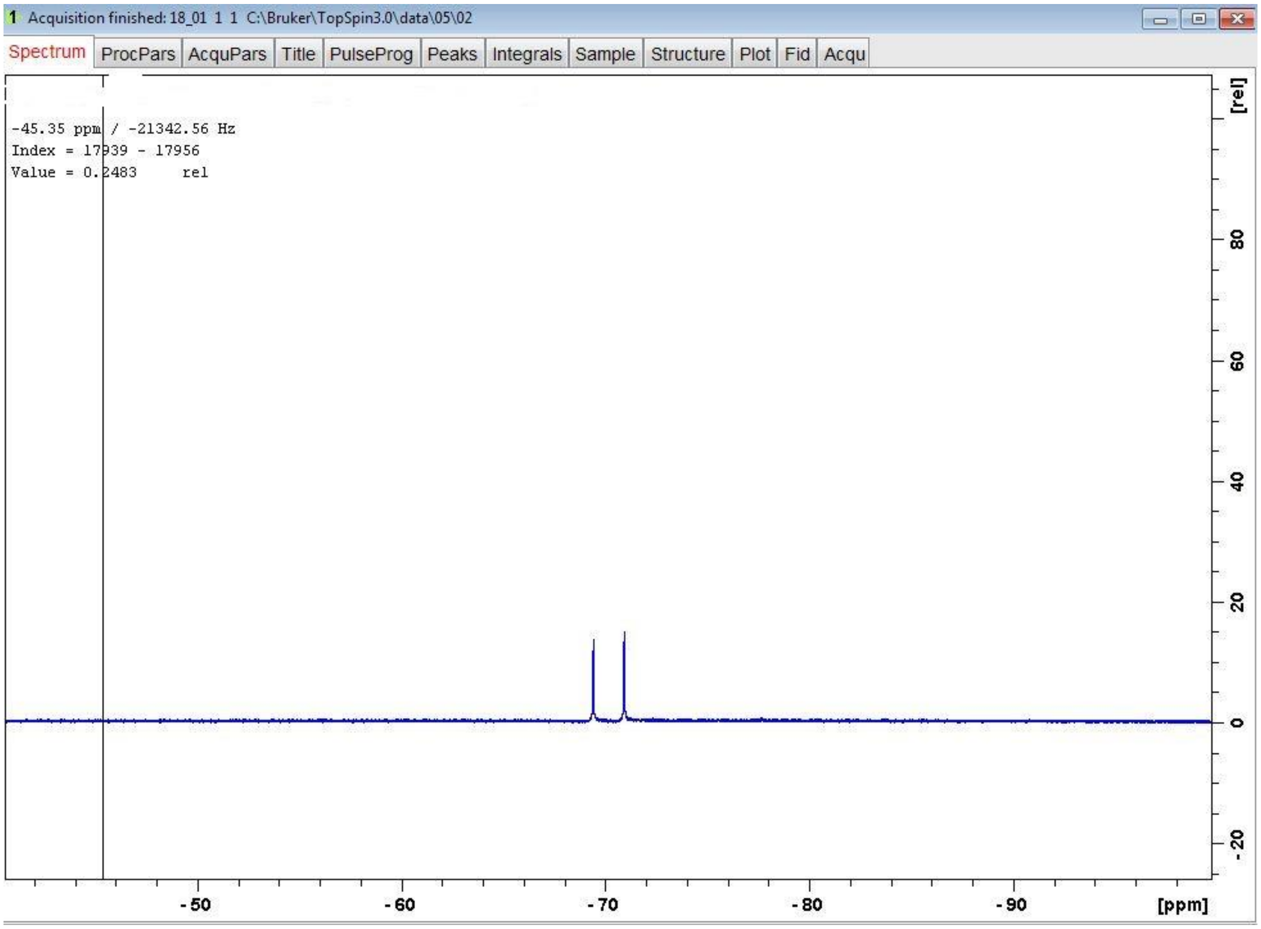

Figure S10 - ^{19}F NMR Spectrum of Poly(1-butyl-2,3-dimethyl-4-vinylimidazolium hexafluorophosphate)
